# Effect of chemical interaction between oleic acid and L-Arginine on oral perception, as a function of polymorphisms of *CD36* and *OBPIIa* and genetic ability to taste 6-*n*-propylthiouracil

**DOI:** 10.1371/journal.pone.0194953

**Published:** 2018-03-22

**Authors:** Melania Melis, Mariano Mastinu, Massimiliano Arca, Roberto Crnjar, Iole Tomassini Barbarossa

**Affiliations:** 1 Department of Biomedical Sciences, Section of Physiology, University of Cagliari, Monserrato, CA, Italy; 2 Department of Chemical and Geological Sciences, University of Cagliari, Monserrato, CA, Italy; Universitat Potsdam, GERMANY

## Abstract

Oral sensitivity to fats varies in individuals influencing nutritional status and health. Variations in oleic acid perception are associated with *CD36* and odorant binding protein (*OBPIIa*) polymorphisms, and 6-*n*-propylthiouracil (PROP) sensitivity, which is mediated by TAS2R38 receptor. L-Arginine (L-Arg) supplementation was shown to modify the perception of the five taste qualities. Here we analyzed the effect of three concentrations (5, 10, 15 mmol/L) of L-Arg on oral perception of oleic acid in forty-six subjects classified for PROP taster status and genotyped for *TAS2R38*, *CD36* and *OBPIIa* polymorphisms. L-Arg supplementation was effective in increasing the perceived intensity of oleic acid in most subjects. The lowest concentration was the most effective, especially in PROP non-tasters or medium tasters, and in subjects with at least an allele A in *CD36* and *OBPIIa* loci. Density Functional Theory (DFT) calculations were exploited to characterize the chemical interaction between L-Arg and oleic acid, showing that a stable 1:1 oleate·ArgH^+^ adduct can be formed, stabilized by a pair of hydrogen bonds. Results indicate that L-Arg, acting as a ‘carrier’ of fatty acids in saliva, can selectively modify taste response, and suggest that it may to be used in personalized dietetic strategies to optimize eating behaviors and health.

## Introduction

Dietary fatty acids play an important role in the regulation of energy and lipid metabolism, and many are their effects on health and illness outcomes of individuals [1]. Consequently, the ability to discriminate dietary fatty acids, selectively and quantitatively, may have crucial implications for nutritional status and human health. Oral sensitivity to dietary fatty acid greatly varies among individuals [2–4]. Therefore, understanding the range of fatty acid oral sensitivity and how it is influenced by genetic and environmental factors may lead to significant insights on the role of taste in fat-rich food intake regulation and metabolism.

Fat taste in humans has been suggested as a sixth primary taste quality by Mattes 2010 [5] and confirmed by others [6–8], but the confirmation will require additional data. The following receptors for taste perception of fatty acids have been shown in rodents and humans: the delayed-rectifying potassium (DRK) channel Kv1.5 [9], the G protein-coupled receptor family (GPR120 and GPR40) [10,11], and the multifunctional CD36 scavenger receptor [8,12], which is expressed on taste bud cells of the circumvallate papillae where it has been shown to initiate the cephalic phase activated by FA perception [12–14]. CD36 expression in taste bud has been found to be reduced in high-fat diet-induced obese rats [15], suggesting that a decreased sensitivity to fat, resulting from a diminished expression of CD36, could lead to an increase of fatty food intake as a compensatory mechanism. In addition, in humans the role of CD36 in orosensory perception and preference of dietary lipids has been well proved [8,16]. Several studies showed that variations in fat perception and obesity are associated with common variants in *CD36* gene [8,16,17]. In particular, several data showed that the polymorphism *rs176166* (A/G), whose allele A is characterized by a decreased protein expression, could explain individual variations in fat orosensory perception [18–20], as well as the different metabolic pattern found between lean and obese subjects [21].

Variations in sensitivity to oleic acid have been related to changes in general taste sensitivity as indicated by differences in the expression of salivary proteins, such as carbonic anhydrase 6 [22], which have been associated with sensitivity to the prototypical taste stimulus, 6-*n*-propylthiouracil (PROP) [23–26]. The importance gained by this stimulus in the fields of nutrition and taste is based on results showing that subjects who perceive PROP as more bitter (PROP super-tasters), compared with those who perceive PROP only at high concentrations or not at all (non-tasters), also present a higher sensitivity to a wide range of oral stimuli of all taste qualities [27–38], which could be explained by a higher density of fungiform papillae on their tongue with respect to other PROP taster groups [24,34,39–42]. The relationships between PROP tasting and perception and preferences for fats have been extensively investigated. PROP non-tasters show a lower ability to discriminate fat and creaminess in fatty foods [43–48]. They also exhibit a higher level of preference for dietary fat [44,47,49–52] and consume more fats and high-energy foods than do tasters [50,53]. However, other authors report inconsistent results [54,55].

Recently, individual differences in oleic acid perception has been reported to be associated with variability in a human odorant-binding protein gene (*OBPIIa*) [56]. Subjects who were homozygous for the A-allele in *rs2590498* polymorphism reported to perceive bitterness when tasting a milkshake containing dilute concentrations of oleic acid. Bitter perception was dramatically reduced after eliminating retronasally perceived odorants. Variants of *OBPIIa* could also explain differences in PROP bitterness perception independently from the genotype of the gene codifying for the specific bitter receptor TAS2R38, which explains most of the PROP phenotypic differences with its allelic diversity [57,58].

Individual differences in taste sensitivity have also been attributed to many other factors including morphology and density of taste papillae [24–26,39–41], physical properties of saliva [59], and its chemical constituents [60–63]. Among them, some proteins belonging to the basic proline-rich protein family (Ps-1 and II-2 proteins) and specific amino acids of their sequence, such as L-Arginine (L-Arg), enhance PROP bitterness perception, depending on their concentration in saliva [61–63]. Besides, recently supplementation with L-Arg has been shown to specifically modify the perception of the five taste qualities, thus suggesting this mechanism as altering taste response related to foods [38].

Based on these considerations and given the nutritional value of dietary lipids, it would be of great interest to find a mechanism to modify the taste response related to fats, which might influence lipids intake, also as a function of the factors involved in individual differences of fat perception. To this aim we analyzed the effect of L-Arg supplementation on the oral perception of oleic acid, as a function of the common variants in *CD36* (*rs1761667*) and *OBPIIa* (*rs2590498)* genes, and PROP-tasting genotype and phenotype of subjects. In order to evaluate possible variations of the multimodal oral perception of oleic acid due to L-Arg administration, subjects were tested by delivering the fat stimulus to the oral cavity in absence of nose clips, as it happens when food is ingested. The effect of three concentration of L-Arg (5, 10, 15 mmol/L), which have already been shown to be effective in modifying taste perception of the five taste qualities, was analyzed for the purpose of identifying the concentration with the greatest effectiveness.

## Materials and methods

### Ethics statement

Subjects were informed about the procedure and the aim of the study and signed an informed consent form. The Ethical Committee of the University Hospital of Cagliari approved the study procedures, which were performed in accordance with the latest revision of the Declaration of Helsinki.

### Subjects

Forty-six non-smoking healthy young Caucasian volunteers (8 men and 38 women, age 26.6 ± 0.79 years) were recruited through public advertisements at the University of Cagliari (Italy). All were originally from Sardinia, Italy. They were normal weight with a body mass index (BMI) ranging from 18.6 to 25.3 kg/m^2^ and experienced no change in body weight larger than 5 kg over the previous 3 months. None were dieting or taking medications that might interfere with oral sensory perception. None had food allergies, or scored high on eating behavior scales (assessed by using the Three-Factor Eating Questionnaire) [64]. Normogeusia for four basic tastes (sweet, sour, salty, and bitter) was verified in all participants by a taste strip test (Burghart Messtechnik, Wedel, Germany). This trial was registered at ClinicalTrials.gov (identifier number: UNICADBSITB-1).

### Experimental protocol

Subjects were tested in three sessions. In the first two (on two consecutive days) subjects were classified for PROP-taster status. In the third session, 1-month later, their oral perception for oleic acid, and its changes due to L-Arg administration were assessed. Subjects were requested to refrain from eating, drinking (except water) and using oral care products for at least 2 hours prior to testing. In women, the assessments were done on the sixth/seventh day of their menstrual cycle to avoid changes of oral sensitivity due to the estrogen phase [65–68]. The testing room was kept virtually free from odors, was lit with standard solar lighting (15,000 lux) and noise level was kept at a minimum. Subjects had to be in the testing room 15 min before the beginning of trials in order to adapt to the environmental conditions (23–24°C, 40–50% relative humidity), which were kept constant during the experimental session. Solutions were prepared in spring water the day before each session and stored in the refrigerator until 1 hour before testing.

A sample (2-mL) of whole mixed saliva was collected from each subject into an acid-washed polypropylene test tube. Samples of saliva were stored at –80°C until molecular analyses were completed, as described below.

### PROP-taster status

In order to classify each subject for PROP-taster status (as PROP super-taster, medium taster, or non-taster), taste intensity ratings were collected, in 2 successive days, by using two different psychophysical procedures: the three-solution test [69], and the impregnated paper screening test [70], which have been validated in several studies [23,25,26,62]. Both procedures are highly reliable as they strongly correlate with the degree of activation of peripheral taste function [71,72]. In the three-solution test, the taste-intensity ratings were collected for three suprathreshold solutions (in 10-mL samples) of PROP (0.032, 0.32, and 3.2 mmol/L) (Sigma-Aldrich, Milan, Italy) and NaCl (0.01, 0.1, 1.0 mol/L) (Sigma-Aldrich, Milan, Italy). Instead, the impregnated paper screening test is based on the ratings of 2 paper disks, one impregnated with PROP solution (50 mmol/L) and the other with NaCl (1.0 mol/L). Stimuli were presented at room temperature. In both tests, taste-intensity ratings for PROP and sodium chloride (NaCl) were collected from each subject by using the Labeled Magnitude Scale (LMS) [73]. LMS gives subjects the freedom to rate the perceived taste intensity of PROP and NaCl, in relation to the ‘strongest imaginable’ oral stimulus they had ever experienced in their life. Each subject was trained in the use of the LMS before testing. In both procedures, PROP and NaCl were presented in a blind and counterbalanced order. Subjects who gave intensity ratings higher to PROP solutions than to NaCl, or rated the PROP disk higher than 67 mm on the LMS, were classified as PROP super-tasters, while those who gave ratings to PROP solutions lower than to NaCl solutions, or rated the PROP disk lower than 13 mm on the LMS were classified as non-tasters. Finally, those who gave comparable ratings to the two stimuli, or rated PROP disk with intermediate ratings, were classified as medium-tasters. Only subjects likewise classified by the two procedures were included in the study. Ten subjects were classified as PROP super-tasters (21.74%); 19 as medium-tasters (41.30%) and 17 as non-tasters (36.96%). Three-way ANOVA was used to validate the presence of the three taster groups (see S1 Table).

### Molecular analysis

DNA was extracted from saliva samples using the QIAamp^®^ DNA Mini Kit (QIAGEN S.r.l., Milan, Italy) according to the manufacturer’s instructions. Purified DNA concentration was estimated by measuring the optical density at 260 nm with an Agilent Cary 60 UV-Vis Spectrophotometer.

Subjects were genotyped for three single nucleotide polymorphisms (SNPs) *rs713598*, *rs1726866*, and *rs10246939*, respectively at base pairs 145 (C/G), 785 (C/T), and 886 (G/A) of the *TAS2R38* locus, that consist of three amino acid substitutions (Pro49Ala, Ala262Val, and Val296Ile), which give rise to two major haplotypes, PAV (the dominant taster variant) and AVI (the non-taster recessive one) and three rare haplotypes (AAI, AAV, and PVI). A polymerase chain reaction (PCR) was employed to amplify the short region of the *TAS2R38* locus, including the first polymorphism of interest (*rs713598*), followed by analysis with restriction enzyme (*Hae*III) of the fragments obtained according to our previous work [24]. The *rs1726866* and *rs10246939* SNPs were determined by using the TaqMan^®^ SNP Genotyping Assay (C_9506827_10 for the *rs1726866* assay and C_9506826_10 for the *rs10246939* assay; Applied Biosystems by Life-Technologies Italia, Europe BV) [74–76] according to the manufacturer’s specifications. Replicates and positive and negative controls were included in all reactions.

Subjects were also genotyped for the single nucleotide polymorphism (SNP), *rs1761667* (G/A), of *CD36*, located at the −31118 promoter region of exon 1A. To genotype the *CD36 rs1761667* polymorphism molecular analyses were carried out by PCR followed by analysis with restriction enzyme (*Hha*I) of the fragments obtained according to Banerjee et al. 2010 [77]. All digestion products were separated by electrophoresis on a 2% agarose gel and the DNA bands were visualized by ethidium bromide staining and ultraviolet light to score the deletion. PCR 50 bp Low Ladder DNA was used as a molecular mass marker (Gene Ruler™ -Thermo Scientific).

Finally, subjects were genotyped for the *OBPIIa* gene polymorphism *rs2590498* (A/G) by using custom TaqMan® SNP Genotyping Assay (Applied Biosystems by Life-Technologies Italia, Europe BV) according to Tomassini et al. 2017 [56], and as briefly described below. The following primers sets were used: the forward GCCAGGCAGGGACAGA and the reverse CTACACCTGAGACCCCACAAG and two TaqMan probes were designed according to the OBPII gene (bold and underlined), probe/reporter 1: VIC-TCGGTGAC**A**TGAACC and probe/reporter 2: FAM–TCGGTGAC**G**TGAACC. Replicates as well as positive and negative controls were included in all reactions.

Molecular analysis at the three SNPs of the *TAS2R38* locus identified 8 subjects who were PAV homozygous, 20 were heterozygous, and 15 were AVI homozygous. Three subjects with rare haplotype were excluded. Molecular analysis at the SNP (*rs1761667*) of the *CD36* identified: 15 subjects who were homozygous GG for *CD36* locus, 23 who were heterozygous, and 8 who were homozygous AA. While, the observed genotype distribution for *OBPIIa* locus identified 9 subjects who were homozygous AA, 14 who were heterozygous, and 23 who were homozygous GG.

### Quantum-mechanical calculations

Theoretical calculations were carried out at the Density Functional Theory (DFT) [78] level with the aim of investigating the relative stabilities of oleic acid in its neutral protonated and anionic deprotonated form, L-Arg, L-argininium (L-ArgH^+^) and the neutral molecular adduct resulting from the interaction of L-Arg and oleic acid (oleic acid·Arg). In DFT methods, the relationship between the electron density and the energy of the molecular system is described by a “functional”, i.e. a functions of another function. Based on the results of our previous calculations on Arg, and a variety of different molecular systems, also including the evaluation of intermolecular interactions, we adopted an hybrid functional (mPW1PW) [79]. Electron shells were mathematically represented by split-valence basis sets, including polarization functions [80–82]. Atomic charges, bond orders, and interaction strengths were calculated at Natural Bond Orbital level (NBO) [83]. Calculations were carried out on isolated molecules (gas phase) and in the presence of solvents, namely water and n-pentadecane to mimic the differently polar environment within and outside oleic acid vesicles. More details on the adopted QM methods are available as S1 File.

### Effect of L-Arg supplementation on oleic acid oral perception

The effect of supplementation with of L-Arg on oleic acid multimodal oral perception was assessed in each subject in the third session. In order to evaluate possible variations in oleic acid perception due to L-Arg administration, an amount (1 μL) of oleic acid just above threshold [17] was also presented supplemented with three concentrations of L-Arg (5, 10 and 15 mmol/L), which had previously been shown to be effective in modulating taste perception of five taste qualities [38,62,63].

Stimuli were presented to each subject, in the absence of nose clips, by means of filter paper disks (1.5 cm diameter) according to Melis et al [17]. After rinsing mouth with spring water, each subject was presented, in a random order and a double blinded way, with 5 filter paper disks impregnated with 15 μL of each stimulus. One contained only mineral oil (10 μL) supplemented with spring water (5 μL); one contained a mixture of oleic acid (1 μL) and mineral oil (9 μL) supplemented with spring water (5 μL); three contained a mixture of oleic acid (1 μL) and mineral oil (9 μL) supplemented with L-Arg (5 μL) at different concentrations. Subjects were instructed to place the paper disk on the center of their tongue, keep it in the mouth to savor it for 10 s in order to facilitate the release of the stimulus, then spit it out. Each stimulation was followed by oral rinsing with spring water. The interstimulus interval was set at 5 min. After 1 h, each subject was presented with three more paper disks as controls which contained mineral oil (10 μL) supplemented with L-Arg (5 μL) at different concentrations. Perceived intensity ratings for each stimulation were collected by having the subject place a mark on the LMS corresponding to his/her stimulus perception.

### Statistical analyses

Fisher’s exact test was used to compare the percentages of subjects who showed an increase of perceived intensity ratings when the oleic acid was supplemented with the three concentrations of L-Arg (5, 10 and 15 mmol/L), also as function of PROP-taster status, or polymorphisms of *TAS2R38*, *CD36 (rs1761667*) and *OBPIIa (rs2590498)* genes. One-way ANOVA was used to compare the perceived intensity ratings according to PROP taster status, or polymorphisms of *TAS2R38*, *CD36 (rs1761667*) and *OBPIIa (rs2590498)* genes. Repeated-measures ANOVA was used to analyze the percentage increase of perceived intensity ratings by subjects in which the three concentration of L-Arg (5, 10 and 15 mmol/L) were effective, also as function of PROP-taster status, or polymorphisms of *TAS2R38*, *CD36 (rs1761667*) and *OBPIIa (rs2590498)* genes. Post hoc comparisons were conducted with the Fisher’s least significant difference (LSD) test, unless the assumption of homogeneity of variance was violated, in which case the Duncan’s test was used. Statistical analyses were conducted using STATISTICA for WINDOWS (version 7; StatSoft Inc, Tulsa, OK, USA) with 95% confidence interval. *P* values < 0.05 were considered significant.

## Results

### Theoretical calculation: Oleic acid/L-Arg interaction

**Fig 1:**
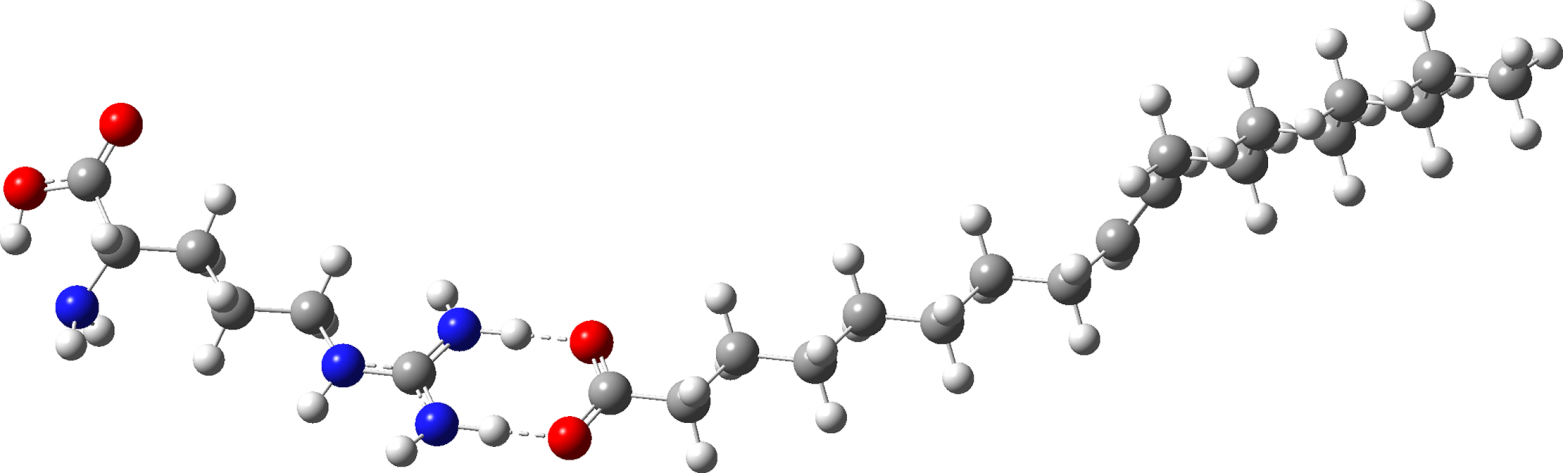
DFT optimized structure of H-bonded adduct between oleic acid (right) and L-ArgH^+^ (left) calculated in water. Oxygen atoms are depicted in red, nitrogen atoms in blue, carbon atoms in grey and hydrogen atoms in white.

Quantum-mechanical (QM) calculations at the DFT level [78] allowed us to optimize the geometry of oleic acid, the oleate anion, L-Arg, and the protonated form of L-Arg (L-ArgH^+^). The interaction of oleic acid and L-Arg, or oleate and L-ArgH^+^, was hypothesized to lead to the hypothetical adduct oleic acid·L-Arg. The formation of this adduct was evaluated in the gas phase, in water, and in an organic solvent (n-pentadecane) featuring the same very low polarity as oleic acid (ε_r_ = 2.033 [84] and 2.03, respectively, at 298 K). In all cases, the adduct features a double hydrogen-bonded system involving the carboxylate group of deprotonated oleic acid and the two terminal NH_2_ groups of L-ArgH^+^ (Fig 1) featuring two O⋯H–N linear systems (average distances, gas phase: C-O, 1.256; N-H, 1.105; O⋯H, 1.461 Å; O–H–N, 178.85°; water: C-O, 1.257; N-H, 1.060; O⋯H, 1.619 Å; O–H–N, 179.05°; n-pentadecane: C-O, 1.257; N-H, 1.084; O⋯H, 1.523 Å; O–H–N, 178.97°). The optimized bond distances and the total electronic energies (*E*) calculated for the free synthons and the adduct in the gas phase, in water, and in the organic phase, show that it is very strong in the limit conditions explored by the calculations (Δ*E*_add_ = -31.81, -34.88, and -32.83 kcal mol^-1^ in vacuo, in water and in n-pentadecane). An NBO analysis was carried out to verify the effect of the solvent variation on the strength of the formed hydrogen bonds, showing that, as expected, the two H-bonds are energetically equivalent (< 5%) and derive from the charge-transfer (CT) interaction of the lone pairs of electrons (LP) on the negatively-charged carboxylate oxygen atoms to the antibonding NBOs localized on the N–H bonds (the average CT varies between 42.86 and 72.99 in water and in the gas phase, respectively).

### Effect of L-Arg supplementation on oleic acid perception

**Fig 2:**
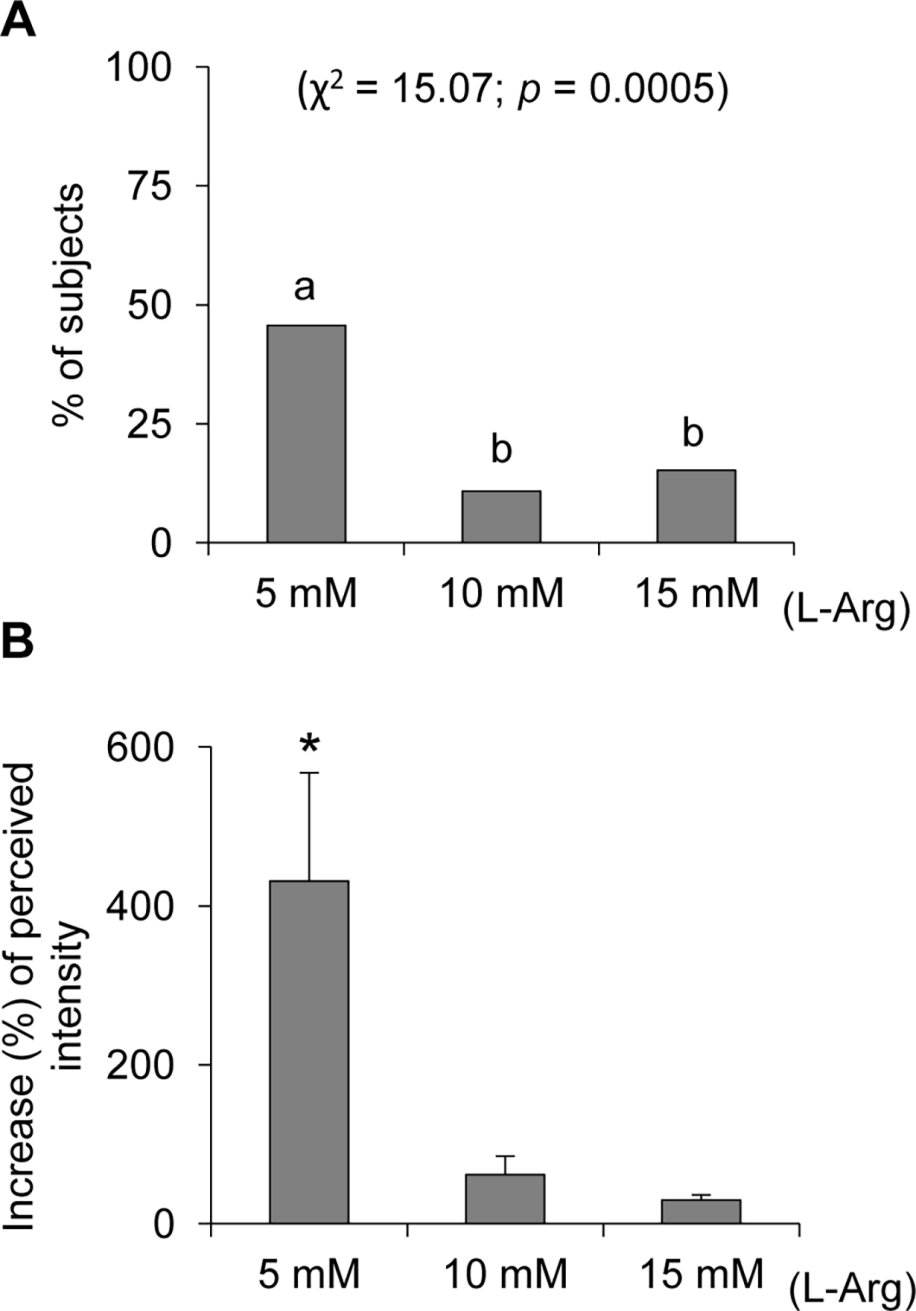
Effect of L-Arginine (L-Arg) supplementation on oleic acid responsiveness. (**A**) Percentage of subjects who showed an effective increase of perceived intensity ratings when the oleic acid was supplemented with three concentrations of L-Arg (5, 10 and 15 mmol/L). (**B**) Percentage increase of perceived intensity ratings by the subjects in which the three concentrations of L-Arg (5, 10 and 15 mmol/L) were effective. n = 46. Different letters on top of bars (a or b) indicate significant differences (*p* ≤ 0.0044; Fisher’s exact test). * = Significant difference with respect to the corresponding value assessed before supplementation (*F*_(1,20)_ = 8.1696, *p* = 0.0097; Repeated-measures ANOVA).

The paper disks containing only mineral oil supplemented with spring water did not evoke any taste perception in all subjects. Differently, most subjects (89.1%) described a perceived intensity between weak and moderate on the LMS (12.0 ± 1.54 mm) when they tested the paper disk impregnated with the mixture of oleic acid and mineral oil supplemented with spring water, while 10.9% (n = 5) could not perceive this stimulus. The supplementation with L-Arg to the mixture of oleic acid and mineral oil was effective in increasing perceived intensity in 72% of subjects, and the individuals who perceived no oleic acid mixture with no L-Arg added, experienced perception for the first time. Fisher’s exact test showed that the percentage of subjects who showed an increase of responsiveness when the oleic acid was supplemented with L-Arg was different at the three concentrations of L-Arg (5, 10 and 15 mmol/L) (χ^2^ = 15.07, *p* = 0.0005) (Fig 2A). Specifically, the lowest concentration (5 mmol/L) was effective in 46% of subjects, while the supplementation with 10 or 15 mmol/L determined only a small increase in the number of subjects who became responsive (11% or 15%, respectively). These later values did not differ statistically from each other (χ^2^ = 0.390, *p* = 0.553).

The percentage increase in the perceived intensity ratings by subjects in which the three concentrations of L-Arg (5, 10 and 15 mmol/L) were effective is shown in Fig 2B. The supplementation with the lowest concentration (5 mmol/L) determined a significant increase in perceived intensity with respect to values determined in response to stimulation with oleic acid with no L-Arg added (*F*_(1,20)_ = 8.169, *p* = 0.0097; Repeated-measures ANOVA), while no significant changes were found after supplementation with 10 and 15 mmol/L of L-Arg in those subjects in which these concentrations were effective (Fig 2B).

The perceived intensity of oleic acid with no L-Arg added, according to PROP taster status of subjects, did not show significant differences (*p* > 0.05), and was as follows: PROP super-tasters showed an intensity of 13.60 ± 3.14 mm in the LMS, medium tasters of 11.70 ± 2.41 mm and non-tasters of 8.53 ± 2.28 mm. The perceived intensity for oleic acid without added L-Arg by subjects genotyped for *CD36* gene did not show significant differences (*p* > 0.05), and was as follows: subjects with genotype GG showed an intensity of 13.83 ± 2.06 mm, heterozygous subjects of 8.00 ± 2.55 mm and genotypes AA of 8.33 ± 4.02 mm. The perceived intensity for oleic acid without added L-Arg by subjects genotyped for *OBPIIa* gene was as follows: subjects with genotype AA showed an intensity of 17.44 ± 3.19 mm, heterozygous subjects of 8.43 ± 2.56 mm and genotypes GG of 9.65 ± 1.99 mm. In this case, the perceived intensity by genotypes AA was higher than that tested by genotypes AG and GG (*p* < 0.041; Duncan test), which gave no different ratings (*p* > 0.05).

**Fig 3:**
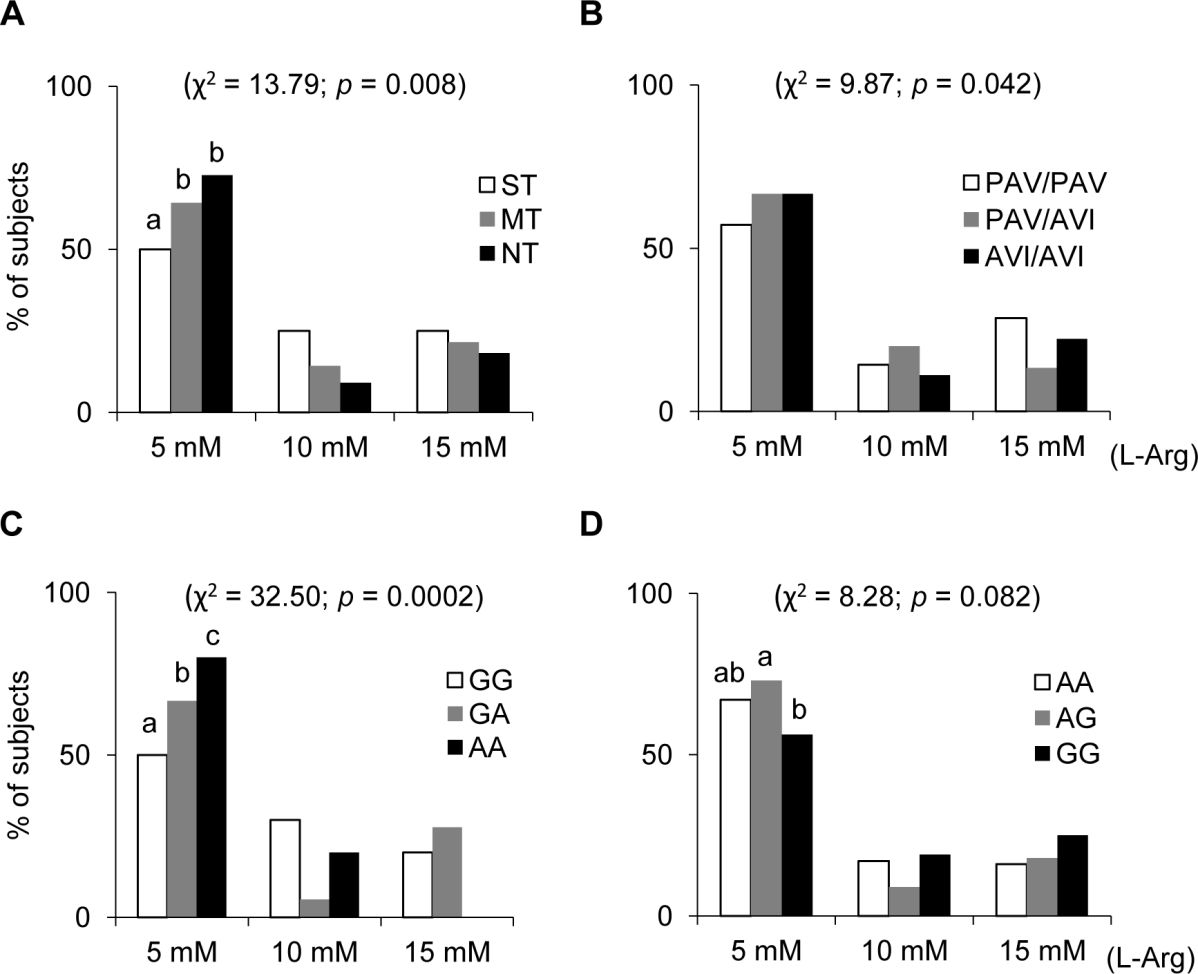
Percentage of subjects who showed an effective increase of responsiveness when oleic acid was supplemented with three concentration of L-Arg (5, 10 and 15 mmol/L). (**A**) Data shown for each PROP taster group. (**B**) Data shown for each genotype of *TAS2R3* gene. (**C**) Data shown for each genotype of *CD36* gene. (**D**) Data shown for each genotype of *OBPIIa* gene. n = 46. Different letters on top of bars (a, b or c) indicate significant differences (*p* ≤ 0.045; Fisher’s exact test).

The percentage of subjects who showed an increase of responsiveness when the oleic acid was supplemented with three concentrations of L-Arg (5, 10 and 15 mmol/L), according to PROP taster status or polymorphisms of *TAS2R38*, *CD36 (rs1761667*) and *OBPIIa (rs2590498)* genes is shown in Fig 3. Fisher’s exact test showed that the percentage of subjects responsive to L-Arg classified as non-tasters, medium tasters or PROP super-tasters was different at the three concentrations (χ^2^ = 13.79, *p* = 0.008) (Fig 3A). Specifically, the percentage of subjects responsive to the lowest concentrations (5 mmol/L) who were classified as non-tasters (73%) or medium tasters (64%) was higher than that of PROP super-tasters (50%) (χ^2^ > 3.99, *p* < 0.045; Fisher’s exact test). No significant changes were found between non-tasters and medium tasters, or in subjects responsive to 10 and 15 mmol/L of L-Arg (*p* > 0.05). Consistent data, although not significant, were found in subjects genotyped for *TAS2R38* gene (Fig 3B). Fisher’s exact test also showed that the percentage of subjects responsive to L-Arg with genotyped GG, GA and AA in *CA36* gene was different at the three concentrations (χ^2^ = 34.497, *p* = 0.00002) (Fig 3C). In particular, the percentage of subjects responsive to the lowest concentrations of L-Arg who had a pair of alleles A (80%) was higher than that of subjects with only one allele A (67%) (χ^2^ = 4.77, *p* = 0.029; Fisher’s exact test), which in turn was higher than that of subjects with homozygous GG genotype (50%) (χ^2^ = 5.95, *p* = 0.0147; Fisher’s exact test). No significant changes related to *CD36* gene were found in subjects responsive to 10 and 15 mmol/L of L-Arg (*p* > 0.05). The percentage of subjects responsive to L_Arg (5 mmol/L) with genotype AA in *OBPIIa* gene (67%) was not different from that of heterozygous (73%), which was higher than that of subjects with homozygous GG genotype (56%) (χ^2^ = 6.31, *p* = 0.012; Fisher’s exact test). No significant changes related to *OBPIIa* genotypes were found in subjects responsive to 10 and 15 mmol/L of L-Arg (*p* > 0.05).

**Fig 4:**
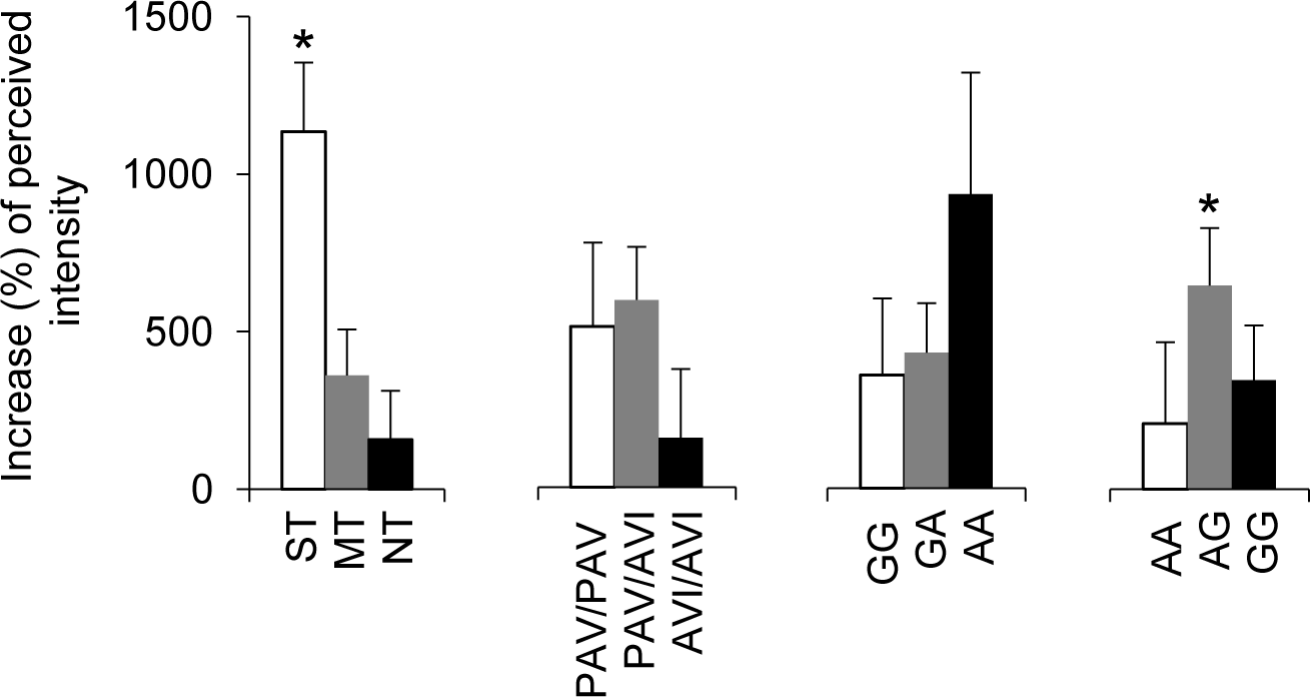
Percentage increase of perceived intensity ratings by the subjects in which L-Arg (5 mmol/L) was effective according to PROP taster status or polymorphisms of *TAS2R38*, *CD36* (*rs1761667*) and *OBPIIa* (*rs2590498*) genes. n = 33. * = Significant difference with respect to the corresponding value assessed before supplementation (*p* = 0.019; Fisher’s LSD test subsequent to repeated-measures ANOVA).

Fig 4 shows the percentage increase of perceived intensity ratings by the subjects in which the lowest concentration of L-Arg (5 mmol/L) were effective according to PROP taster status or polymorphisms of *TAS2R38*, *CD36* (*rs1761667*) and *OBPIIa* (*rs2590498*) genes. Responsive subjects classified as PROP super-tasters showed a significant increase of perceived intensity, with respect to that perceived in response to oleic acid alone (*p* = 0.0018; Fisher’s LSD test subsequent to repeated-measures ANOVA). No significant changes of perceived intensity were found in non-tasters or medium tasters (*p* > 0.05). No significant changes were also found when data were analyzed according to polymorphisms in *TAS2R38* or *CD36* gene, although subjects who carried genotype AA in *CD36* gene showed an increase of perceived intensity higher than GG and GA genotypes. In addition, responsive subjects who were heterozygous for *OBPIIa* gene showed a significant change of perceived intensity, with respect to that perceived in response to oleic acid alone (*p* = 0.019; Fisher’s LSD test subsequent to repeated-measures ANOVA), while no significant changes were found in subjects with AA or GG genotype.

L-Arg did not evoke significant changes in oral perception of oleic acid in 13 subjects (28%) who were not characterized by a particular *CD36* or *OBPIIa* genotype or PROP taster status.

The paper disks containing mineral oil supplemented with l-Arg (5 mmol/L and 10 mmol/L) did not evoke taste perception in all subjects. Differently, the paper disks containing mineral oil supplemented with l-Arg (15 mmol/L) were described as weakly bitter in LMS (4.74 ± 1.01 mm) in 70% of subjects, while the remainder (30%) were not able to identify any of the tastes.

No harms or unintended effects were observed.

## Discussion

There is some evidence that a link exists between accumulation of adiposity and reduced chemosensory functions, such as decreased oral fat sensation [85–91], sweet taste [92], salty and bitter taste [93], umami taste [94] and general taste and smell capacity [95]. However, other authors report inconsistent data [96,97], which could be due to the presence of confounding variables [98–100]. In any case, an optimal body composition, favourable to fulfil physiological needs, depends on the balance between energy expenditure and food intake, which is in turn regulated by an adequate food choice governed by individual taste sensitivity [101–103]. Given the nutritional value of dietary fats [1] and the ample individual variations shown in perception, preference for, and consumption of lipids [16,104], the present work provides new insights toward the identification of a mechanism capable of modifying orosensory responses related to fats, also as function of factors implied in individual differences in fat perception.

Our results show that oral supplementation with L-Arg was effective in increasing the perception of oleic acid. The role of L-Arg in taste function and its versatility in modifying taste responses has been extensively underscored. It has been described as a salivary component that contributes to individual differences in PROP bitterness perception, depending on its concentration in saliva [61–63]. Besides, its supplementation has been shown to enhance the responsiveness to bitter compounds, such as PROP or caffeine [38,62,63], the perception of which is mediated by different bitter receptors [58,105]. This suggests that the facilitating action of L-Arg is due to an increase in the availability of molecules at receptor sites, rather than to the binding of molecules with the specific receptor [63]. L-Arg is also known to suppress the bitter taste of quinine by specifically blocking the T2R4 receptor [106–108] or to determine profound modification of perception of other taste qualities [38]. Our results extend the knowledge to the fat taste, by demonstrating the L-Arg supplementation increases perception of oleic acid by affecting both the number of subjects who can perceive an amount of oleic acid which previously resulted to be just above threshold [17] and the intensity of sensation perceived. The results also show that L-Arg was more effective when presented at the lowest test concentration used, thus suggesting that a small surplus of this amino acid in saliva is sufficient to determine a significant increase in the perception of oleic acid, while further amounts seem be in excess. However, our data do not allow to judge which concentration range may actually have the strongest effect. The fact that the stimulation with the most effective concentration of L-Arg (used as a control) was tasteless, rules out the possibility that L-Arg contributes to the perception enhancement of oleic acid.

In addition, our results show that the modifications of perception of oleic acid induced by L-Arg administration are related to the PROP taster status of subjects and common variants in *CD36* (*rs1761667*) and *OBPIIa* (*rs2590498)* genes. In particular, supplementation of L-Arg at the lowest concentration determined an increase in the number of subjects who could perceive oleic acid, mostly in PROP non-tasters and medium tasters. These results, which cannot be explained by variations found in relation to *TAS2R38* gene, are in accordance with previous data showing that L-Arg enhanced PROP bitterness perception in PROP non-tasters and medium tasters who had low levels of this amino acid in their saliva, as compared to PROP super-tasters [63]. However, the fact that PROP super-tasters showed a greater increase of perceived intensity ratings is not surprising given the fact that these individuals have a higher density of fungiform papillae on their tongue, as compared with other PROP taster groups [24,34,39–42]. Therefore, by considering that the facilitating action of L-Arg is due to an increase in the availability of molecules at receptor sites as previously shown [63], in PROP super-tasters who have a higher density of papillae, L-Arg can facilitate the activation of a higher number of taste receptors.

Interestingly, the analysis of data according to polymorphism, *rs1761667* in *CD36* indicate that the effectiveness of L-Arg supplementation in increasing the perception of oleic acid is directly related to the presence of allele A in *rs1761667* polymorphism of *CD36* gene, which has been associated with a lower expression of CD36 scavenger receptor, with respect to that of allele G [109,110]. In fact, L-Arg was more effective in increasing the number of subjects who could perceive oleic acid, when they had an allele A in *CD36* locus, with respect to subjects carrying the allele G; besides, having a pair of alleles A further increased the number of responding subjects and their degree of responsiveness, although the latter not significantly.

Surprisingly, our data showed that the presence of a single taster variant in the human odorant-binding protein gene (*OBPIIa*) resulted to be the most favorable condition for having the strongest effect of L-Arg in increasing oleic acid perception. In fact, the percentage of responsive subjects with genotype homozygous AA, which has been previously associated to an increased oleic acid perception [56], was not different from that of heterozygous, which was higher than that of subjects with homozygous genotype for the non-taster variant (GG). In addition, responsive heterozygous gave higher perceived intensity ratings than genotypes AA or GG. These results seem to suggest that the effect of L-Arg on the olfactory component of perception of oleic acid is greater in the presence of a single taster variant of the odorant-binding protein, while the supplementation of L-Arg in subjects with a pair of alleles AA did not give further advantages.

Our results also confirm previous evidence [17] showing that the paper screening test is an effective and quick method for assessing the multimodal oral perception of dietary fats when they are presented in a mixture with mineral oil which does not evoke taste perception.

Quantum-mechanical DFT calculations were carried out to get an insight on the nature of the interaction between L-Arg and oleic acid. L-Arg is a basic amino acid (pK_r_ = 12.48) that remains protonated at the guanidine residue, both in the free form, in proteins [111,112], and lipid membranes [113], across the entire physiological pH range. Oleic acid, as a long chain fatty acid, spontaneously forms vesicles at a pH close or lower to its pK_a_ value [114]. The pK_a_ value of carboxylic acids is generally close to 4.8 [115], but self-association processes reduce the apparent value of the acidity constants [116]. In the case of oleic acid, an apparent value of pK_a_ (pK_app_) of 9.85 was reported [117]. The acidity of oleic acid and the basicity of L-Arg suggest that at physiological pH they are in largely present as oleate and L-argininium, respectively. Since L-ArgH^+^ was proved to form a H-bonded adduct with PROP, we have turned to study the nature of the interaction between the oleate anion and L-ArgH^+^ cation or, which stoichiometrically is the same, between L-Arg and oleic acid (oleic acid ·L-Arg). The optimization of the geometry of oleic acid ·L-Arg carried out at DFT level shows that the adduct is stabilized by Δ*E*_add_ ≈ 30 kcal mol^–1^ with respect to the free synthons, due to the formation of two strong H-bond O⋯H–N interactions. This type of interaction is well-known in the literature, and more than 50 structures have been deposited at the Cambridge Structural Database showing L-ArgH^+^ interacting with carboxylic acids by means of the same–CO_2_⋯(H_2_N)_2_C< 8-membered ring motif, such as the complex of L-Arg and pimelic acid [118]. Since the carbonyl groups of oleic acid are exposed to water in the vesicles, calculations were extended to two limit solvation conditions. Implicit solvation calculations were carried out in water and n-pentadecane, a solvent featuring the same polarity as that reported for oleic acid at 298 K (ε_r_ = 2.033) [119], therefore mimicking the L-Arg/oleic acid interaction at the interface and between the vesicles, respectively. Interestingly, notwithstanding a large variation of the H-bonds strengths evaluated by a SOPT analysis, in both solvents the results of the gas phase were confirmed, with stabilization energies of about -35 and -33 kcal mol^-1^. Since the carboxyl group is important for the taste cues of fatty acids [120,121], it is conceivable that the stability of the adduct is possibly responsible for the transport of free oleic acid molecules from polydispersed vesicles to saliva in the oral cavity. In this sense, the role of L-Arg in increasing the taste sensitivity to oleic acid could be analogous to that previously hypothesized in increasing the bitter-sensitivity to PROP [63]. Remarkably, since the interaction with L-Arg only involves the carboxylate–COO–group of the acid, the same mechanism in principle could be extended to different long-chain fatty acid and L-Arg could induce an enhancement in their perception.

## Conclusions

The present results further elucidate the role of L-Arg supplementation in modifying taste responses related to foods highlighting its facilitating effects on oral perception of oleic acid, the major fatty acid in human diet [122], also as a function of the factors implied in individual taste differences, and hence food preferences. Therefore, the use of L-Arg administration may be an helpful tool in designing efficient personalized nutritional strategies aimed at optimizing feeding behaviors and health.

## Supporting information

S1 Table Ratings of perceived taste intensity in response to three concentrations of PROP and NaCl in the PROP taster groups. Values are means ± SEM. n = 46. Three-way ANOVA was used to compare PROP bitterness intensity ratings with NaCl saltiness intensity ratings across groups (F_(4,258)_ = 5.199; *p* = 0.00048). * Significant difference between PROP and the corresponding NaCl concentration (*p* < 0.0015; Newman Keuls test).
(PDF)

S1 File Quantum-mechanical calculations. (PDF)

S2 File Molecular analysis at polymorphisms *rs713598* of *TAS2R38* locus and *rs1761667* of *CD36* gene. (PDF)

S1 Data set Molecular analysis at polymorphisms *rs1726866*, and *rs10246939* of *TAS2R38* and *rs2590498* of *OBPIIa* gene. Results of the TaqMan^®^ SNP Genotyping Assay.
(XLS)
