# Supplementary material for: Effect of chemical interaction between oleic acid and L-Arginine on oral perception, as a function of polymorphisms of CD36 and OBPIIa and genetic ability to taste 6-n-propylthiouracil
Source: PLoS One. 2018 Mar 22;13(3):e0194953. doi: 10.1371/journal.pone.0194953 (PMC5864069; doi:10.1371/journal.pone.0194953)
Supplement: S1 Table — Values are means ± SEM. n = 46. Three-way ANOVA was used to compare PROP bitterness intensity ratings with NaCl saltiness intensity ratings across groups (F(4,258) = 5.199; p = 0.00048). * Significant difference between PROP and the corresponding NaCl concentration (p < 0.0015; Newman Keuls test). (PDF) [file pone.0194953.s001.pdf]

S1 Table. Ratings of perceived taste intensity in response to three concentrations of PROP and NaCl in the taster groups.

	**Super-tasters (n = 10)**	**Medium-tasters (n = 19)**	**Non-tasters (n = 17)**
**PROP (mmol/L)**			
0.032	10.72 ± 2.38	4.38 ± 1.31	0.83 ± 0.41
0.32	54.83 ± 6.16 *	37.07 ± 3.14	4.74 ± 1.06 *
3.2	92.00 ± 2.90 *	60.31 ± 3.84	25.11 ± .69 *
**NaCl (mol/L)**			
0.01	2.04 ± 0.74	7.56 ± 2.01	1.96 ± 0.62
0.1	25.45 ± 6.09 *	29.96 ± 4.06	23.76 ± 3.43 *
1	63.27 ± 7.05 *	63.35 ± 5.06	53.90 ± 4.38 *

Values are means ± SEM. n = 46. Three-way ANOVA was used to compare PROP bitterness intensity ratings with NaCl saltiness intensity ratings across groups ($F_{(4,258)}$ = 5.199; p = 0.00048). * Significant difference between PROP and the corresponding NaCl concentration (p < 0.0015; Newman Keuls test).
