# Supplementary material for: Effect of chemical interaction between oleic acid and L-Arginine on oral perception, as a function of polymorphisms of CD36 and OBPIIa and genetic ability to taste 6-n-propylthiouracil
Source: PLoS One. 2018 Mar 22;13(3):e0194953. doi: 10.1371/journal.pone.0194953 (PMC5864069; doi:10.1371/journal.pone.0194953)
Supplement: S1 File — (PDF) [file pone.0194953.s002.pdf]

S1 File. Quantum-Mechanical Calculations

Theoretical calculations were carried out at DFT level [1] on oleic acid, the oleate anion, L-Arginine (L-Arg), the protonated form of L-Arg (L-ArgH$^+$), and the hypothetical adduct oleic acid·L-Arg. All calculations adopted the mPW1PW hybrid functional reported by Adamo and Barone [2], paralleled by split-valence basis sets, including polarization functions, by Schäfer and coworkers [3] in the newer formulations proposed by Weigend (def2svp) [4,5]. The metric parameters for all the compounds were calculated at their fully relaxed geometries, evaluated by means of analytically calculated gradients, by means of the Berny algorithm using GEDIIS [6].

Atomic charges were calculated at Natural Bond Orbital level (NBO) [7] at the optimized geometries. Calculations were also carried out in the presence of water (ε_r = 78.355) and n-pentadecane (ε_r = 2.033) to mimic the differently polar environment within and outside oleic acid vesicles (Tables S2-S16). Solvation was implicitly taken into account by the Polarizable Continuum Model in its Integral Equation Formalism variant (IEF-PCM) Self-Consistent Reaction Field (SCRF) [8]. The adduct formation energy (ΔE_{add}) was evaluated by considering the total electronic energies of oleic acid ($E_{oleic\ acid}$), L-Arg ($E_{L\text{-}Arg}$) and the adduct ($E_{oleic\ acid\cdot L\text{-}Arg}$), according to the relation $\Delta E_{add} = E_{oleic\ acid\cdot L\text{-}Arg} - E_{oleic\ acid} - E_{L\text{-}Arg}$. A Second-Order Perturbation Theory analysis of the Fock matrix in NBO basis (SOPT) was carried out to evaluate the contributions of the hydrogen bonds to the adduct formation in oleic acid·L-Arg.

All calculations were performed with the Gaussian 09 suite of programs [9] on a IBM x3755 server equipped with four 12-core AMD Opteron processors and 64 Gb of RAM and running a 64 bit version of a Linux operating system. The programs Gaussview 5.0.9 [10] and Molden 5.7 [11] were used to investigate the charge distributions and molecular orbital shapes.

References

1. Koch W, Holthausen MC A Chemist's Guide to Density Functional Theory; ed n, editor. Wiley-VCH, Weinheim, Germany 2002.
2. Adamo C, Barone V Exchange functionals with improved long-range behavior and adiabatic connection methods without adjustable parameters: The mPW and mPW1PW models. J Chem Phys. 1998; 108: 664-675.
3. Schäfer A, Horn H, Ahlrichs R Fully optimized contracted Gaussian basis sets for atoms Li to Kr. J Chem Phys. 1992; 97: 2571-2577.
4. Weigend F Accurate Coulomb-fitting basis sets for H to Rn. Phys Chem Chem Phys. 2006; 8: 1057-1065.
5. Weigend F, Ahlrichs R Balanced basis sets of split valence, triple zeta valence and quadruple zeta valence quality for H to Rn: Design and assessment of accuracy. Phys Chem Chem Phys. 2005; 7: 3297-3305.
6. Li X, Frisch MJ Energy-Represented Direct Inversion in the Iterative Subspace within a Hybrid Geometry Optimization Method. Journal of Chemical Theory and Computation. 2006; 2: 835-839.
7. Reed AE, Weinstock RB, Weinhold F Natural population analysis. J Chem Phys. 1985; 83: 735-746.
8. Tomasi J, Mennucci B, Cammi R Quantum Mechanical Continuum Solvation Models. Chem Rev. 2005; 105: 2999-3094.
9. Frisch MJ, Trucks GW, Schlegel HB, Scuseria GE, Robb MA, et al. Gaussian, Inc., Wallingford CT, Gaussian 09, Revision A.02. 2009.
10. Dennington R, Keith T, Millam J Semichem Inc., Shawnee Mission KS, GaussView, Version 5. 2009.
11. Schaftenaar G, Noordik JH Molden: a pre- and post-processing program for molecular and electronic structures*. J Comput Aided Mol Des. 2000; 14: 123-134.

Table S2. Geometry of L-Arg in the orthogonal Cartesian coordinate format optimized in the gas phase at DFT level.

C	1.02545	-0.35289	-0.03585
C	-0.25437	0.42622	0.23008
C	-1.50629	-0.40994	0.01174
N	-2.69777	0.36789	0.23735
C	-3.95397	-0.13835	-0.05871
N	-4.97057	0.71957	0.34307
C	2.29396	0.44971	0.23187
C	3.51518	-0.44711	0.11840
O	4.64081	0.25585	-0.07552
N	2.41039	1.58507	-0.66184
O	3.51479	-1.64179	0.24036
N	-4.23601	-1.24849	-0.62409
H	2.29083	0.73695	1.31044
H	1.04917	-0.68632	-1.08516
H	1.05575	-1.25937	0.58595
H	-0.24726	0.80336	1.26922
H	-0.31325	1.30844	-0.42718
H	-1.52187	-0.77265	-1.02872
H	-1.46530	-1.31237	0.65505
H	-2.67837	0.95709	1.06153
H	-3.41060	-1.83240	-0.73958
H	-4.81154	1.69442	0.11417
H	-5.87245	0.39133	0.01850
H	5.36728	-0.38455	-0.10942
H	1.72444	2.29423	-0.42088
H	3.32637	2.01533	-0.56936

Table S3. Geometry of L-Arg in the orthogonal Cartesian coordinate format optimized in water at IEF-PCM SCRF DFT level.

C	1.02506	-0.35072	-0.04130
C	-0.25568	0.42727	0.22431
C	-1.50237	-0.41818	0.01486
N	-2.69853	0.35977	0.22606
C	-3.94974	-0.13414	-0.06360
N	-4.97366	0.69727	0.35429
C	2.29386	0.44810	0.24131
C	3.51627	-0.44568	0.12099
O	4.62457	0.24086	-0.15821
N	2.40574	1.60483	-0.62993
O	3.51898	-1.63726	0.30719
N	-4.22204	-1.24795	-0.64751
H	2.28620	0.71864	1.32201
H	1.05014	-0.68173	-1.09227
H	1.04811	-1.25796	0.57932
H	-0.24597	0.81112	1.25929
H	-0.31791	1.30506	-0.43825
H	-1.51048	-0.80167	-1.01860
H	-1.46414	-1.30550	0.67717
H	-2.65907	1.03051	0.98435
H	-3.37624	-1.79863	-0.78218
H	-4.79536	1.69157	0.26907
H	-5.87205	0.43162	-0.03009
H	5.36756	-0.38340	-0.17582
H	1.69721	2.28776	-0.37268
H	3.29873	2.06639	-0.47295

Table S4. Geometry of L-Arg in the orthogonal Cartesian coordinate format optimized in the n-pentadecane at IEF-PCM SCRF DFT level.

C	1.02453	-0.34909	-0.03999
C	-0.25508	0.43202	0.22155
C	-1.50496	-0.40813	0.00762
N	-2.69879	0.36938	0.22763
C	-3.95315	-0.13787	-0.05802
N	-4.97266	0.70947	0.35158
C	2.29436	0.44988	0.23294
C	3.51438	-0.44895	0.12109
O	4.63775	0.24785	-0.08621
N	2.41451	1.59006	-0.65604
O	3.50987	-1.64420	0.25543
N	-4.23053	-1.25232	-0.62449
H	2.28873	0.73436	1.31156
H	1.04999	-0.68472	-1.08877
H	1.04942	-1.25414	0.58391
H	-0.24832	0.81458	1.25819
H	-0.31353	1.31032	-0.44086
H	-1.51770	-0.77932	-1.02992
H	-1.46440	-1.30416	0.65904
H	-2.67177	0.98643	1.03091
H	-3.39594	-1.82187	-0.74778
H	-4.80948	1.69268	0.16567
H	-5.87512	0.39882	0.01209
H	5.36974	-0.38742	-0.11627
H	1.72417	2.29441	-0.41123
H	3.32508	2.02759	-0.54205

Table S5. Geometry of L-ArgH$^+$ in the orthogonal Cartesian coordinate format optimized in the gas phase at DFT level.

C	-1.07545	0.24568	-0.19894
C	-2.36296	-0.43076	0.26472
N	-2.75430	-1.65494	-0.41134
C	0.19630	-0.51989	0.13831
C	1.43427	0.23619	-0.31868
N	2.65582	-0.47737	0.04378
C	3.87939	0.04171	0.03087
N	4.07671	1.28139	-0.41658
N	4.91735	-0.67461	0.47180
C	-3.52731	0.58455	0.20267
O	-3.37038	1.75453	0.41507
O	-4.68940	0.02281	-0.06828
H	-2.27061	-0.66632	1.33925
H	-1.13723	0.41777	-1.28751
H	-1.05896	1.24300	0.26474
H	0.25646	-0.68207	1.22729
H	0.17778	-1.51597	-0.33715
H	1.39920	0.38912	-1.41097
H	1.44898	1.22697	0.16285
H	2.56547	-1.46032	0.27146
H	4.78610	-1.56928	0.92302
H	5.86686	-0.34103	0.37906
H	-2.54534	-1.64391	-1.40692
H	-2.36961	-2.50022	-0.00373
H	-4.48232	-0.92423	-0.23781
H	4.97668	1.73433	-0.33610
H	3.33025	1.80730	-0.84908

Table S6. Geometry of L-ArgH$^+$ in the orthogonal Cartesian coordinate format optimized in water at IEF-PCM SCRF DFT level.

C	-1.07435	0.34219	-0.14955
C	-2.34002	-0.38232	0.29798
N	-2.64218	-1.63527	-0.38209
C	0.19920	-0.42645	0.17022
C	1.44464	0.30062	-0.30864
N	2.63757	-0.45142	0.04252
C	3.87933	0.02875	0.01965
N	4.11226	1.26210	-0.42196
N	4.88950	-0.72581	0.44973
C	-3.57385	0.52895	0.18777
O	-3.57705	1.69890	0.48507
O	-4.64147	-0.11940	-0.23451
H	-2.25635	-0.59712	1.37676
H	-1.13846	0.53812	-1.23344
H	-1.06038	1.32507	0.34297
H	0.27260	-0.58867	1.25752
H	0.16198	-1.42322	-0.29882
H	1.39476	0.45330	-1.40013
H	1.49628	1.29353	0.16498
H	2.52265	-1.43103	0.27266
H	4.72228	-1.61242	0.90451
H	5.84525	-0.40351	0.39431
H	-2.26210	-1.66013	-1.32659
H	-2.29554	-2.45072	0.11382
H	-4.27721	-1.03547	-0.41880
H	5.04130	1.65848	-0.39602
H	3.39134	1.80023	-0.88021

Table S7. Geometry of L-ArgH$^+$ in the orthogonal Cartesian coordinate format optimized in n-pentadecane at IEF-PCM SCRF DFT level.

C	-1.07471	0.28599	-0.19335
C	-2.34961	-0.40819	0.27655
N	-2.71150	-1.64793	-0.39258
C	0.19839	-0.48615	0.12037
C	1.44097	0.26138	-0.33683
N	2.64775	-0.46900	0.02798
C	3.87863	0.03604	0.03257
N	4.09634	1.27267	-0.40931
N	4.89768	-0.69592	0.48590
C	-3.54450	0.56427	0.20444
O	-3.44990	1.73797	0.44643
O	-4.67519	-0.03741	-0.11302
H	-2.25014	-0.63222	1.35266
H	-1.14879	0.47258	-1.27860
H	-1.05063	1.27547	0.28532
H	0.26727	-0.66419	1.20619
H	0.16935	-1.47568	-0.36678
H	1.40678	0.41842	-1.42865
H	1.46772	1.25156	0.14473
H	2.54650	-1.45161	0.25108
H	4.74326	-1.58032	0.94959
H	5.85042	-0.36410	0.42942
H	-2.45251	-1.65029	-1.37687
H	-2.32150	-2.47688	0.04356
H	-4.40704	-0.97430	-0.28777
H	5.00532	1.70705	-0.33049
H	3.36783	1.80120	-0.86712

Table S8. Geometry of oleic acid in the orthogonal Cartesian coordinate format optimized in the gas phase at DFT level.

C	-1.80023	3.63176	0.48980
H	-2.33979	4.23848	1.22685
C	-0.46652	3.74900	0.46605
H	-0.01379	4.44493	1.18253
C	0.50916	3.03265	-0.41657
H	1.03143	3.77303	-1.04925
H	-0.01227	2.35665	-1.11069
C	1.55663	2.24482	0.37309
H	2.07800	2.92645	1.06728
H	1.04446	1.50493	1.01113
C	2.58096	1.53914	-0.50670
H	3.08444	2.28164	-1.15004
H	2.05896	0.85337	-1.19661
C	3.62901	0.76104	0.27990
H	3.12337	0.02768	0.93229
H	4.15953	1.44900	0.96081
C	4.64396	0.03959	-0.59808
H	5.14876	0.76922	-1.25372
H	4.11754	-0.65571	-1.27447
C	5.69811	-0.72791	0.19192
H	5.17308	-1.44141	0.85537
H	6.23463	-0.03197	0.85809
C	6.69706	-1.46106	-0.69835
H	7.16206	-0.77317	-1.41683
H	6.17405	-2.22442	-1.30059
C	7.84047	-2.16288	0.01089
O	7.53199	-2.75458	1.18025
H	6.60654	-2.59486	1.39841
O	8.95155	-2.22982	-0.42663
C	-2.66843	2.75977	-0.36434
H	-2.07358	2.24492	-1.13387
H	-3.38150	3.40070	-0.91325
C	-3.46331	1.72843	0.43996
H	-2.75879	1.06464	0.96902
H	-4.03606	2.24415	1.23015
C	-4.41379	0.89444	-0.41053
H	-3.84355	0.39644	-1.21430
H	-5.12537	1.56461	-0.92393
C	-5.18795	-0.15376	0.37938
H	-5.74827	0.34185	1.19144
H	-4.47501	-0.83075	0.88177
C	-6.15196	-0.97623	-0.46760
H	-6.87118	-0.29920	-0.96099
H	-5.59377	-1.46303	-1.28656
C	-6.91538	-2.03478	0.31876
H	-7.46754	-1.54950	1.14287
H	-6.19606	-2.71667	0.80578
C	-7.88819	-2.85043	-0.52545
H	-8.60764	-2.16752	-1.00865
H	-7.33603	-3.33116	-1.35098
C	-8.64315	-3.90818	0.26603
H	-9.23360	-3.45535	1.07726
H	-9.33687	-4.47419	-0.37219
H	-7.95330	-4.63105	0.72800

Table S9. Geometry of oleic acid in the orthogonal Cartesian coordinate format optimized in water at IEF-PCM DFT level.

C	-1.78660	3.56802	0.47419
H	-2.33288	4.18221	1.20075
C	-0.45138	3.68491	0.45875
H	-0.00336	4.38701	1.17280
C	0.53094	2.96490	-0.41401
H	1.05384	3.70473	-1.04689
H	0.01562	2.28337	-1.10736
C	1.58057	2.18661	0.38247
H	2.09332	2.87400	1.07744
H	1.07303	1.44067	1.01750
C	2.61399	1.49008	-0.49408
H	3.10868	2.23807	-1.13793
H	2.10009	0.79628	-1.18199
C	3.67133	0.72673	0.29429
H	3.17693	-0.01334	0.94729
H	4.19429	1.42329	0.97232
C	4.69370	0.01805	-0.58536
H	5.18065	0.75332	-1.24832
H	4.17615	-0.69301	-1.25179
C	5.76545	-0.72358	0.20498
H	5.26241	-1.44406	0.87501
H	6.29646	-0.01274	0.85908
C	6.76699	-1.44195	-0.69514
H	7.20468	-0.75055	-1.42712
H	6.25167	-2.22527	-1.27771
C	7.92647	-2.11249	0.00328
O	7.65574	-2.72749	1.16212
H	6.72399	-2.62432	1.39778
O	9.04825	-2.14168	-0.43334
C	-2.64896	2.68828	-0.37868
H	-2.04245	2.12875	-1.10662
H	-3.32664	3.32644	-0.97421
C	-3.50032	1.71079	0.43491
H	-2.83505	1.05471	1.02176
H	-4.09530	2.27410	1.17463
C	-4.43130	0.85967	-0.41997
H	-3.83524	0.30350	-1.16457
H	-5.09504	1.52163	-1.00337
C	-5.27703	-0.12230	0.38184
H	-5.87151	0.43366	1.12795
H	-4.61223	-0.78464	0.96364
C	-6.21029	-0.97165	-0.47296
H	-6.87603	-0.30886	-1.05330
H	-5.61561	-1.52538	-1.22065
C	-7.05461	-1.95656	0.32659
H	-7.64898	-1.40341	1.07521
H	-6.38910	-2.62046	0.90624
C	-7.98909	-2.80512	-0.52844
H	-8.65282	-2.14000	-1.10698
H	-7.39369	-3.35563	-1.27681
C	-8.82846	-3.78696	0.27617
H	-9.46320	-3.26399	1.00812
H	-9.48966	-4.38089	-0.37151
H	-8.19326	-4.49041	0.83636

Table S10. Geometry of oleic acid in the orthogonal Cartesian coordinate format optimized in n-pentadecane at IEF-PCM DFT level.

C	-1.78852	3.58683	0.48435
H	-2.33860	4.19169	1.21541
C	-0.45508	3.71358	0.47035
H	-0.01296	4.41281	1.19042
C	0.53265	3.00750	-0.40731
H	1.06558	3.75791	-1.01890
H	0.02092	2.34477	-1.12130
C	1.56796	2.20466	0.38333
H	2.06972	2.87008	1.10708
H	1.04780	1.44326	0.98882
C	2.61583	1.53171	-0.49449
H	3.13194	2.29795	-1.09880
H	2.11327	0.86883	-1.22033
C	3.64737	0.72948	0.28946
H	3.13061	-0.03607	0.89427
H	4.15135	1.39227	1.01409
C	4.69449	0.05628	-0.58896
H	5.21291	0.81957	-1.19369
H	4.19523	-0.61108	-1.31239
C	5.72977	-0.73979	0.19734
H	5.19274	-1.49282	0.80426
H	6.23663	-0.07368	0.91500
C	6.76531	-1.41418	-0.69785
H	7.24415	-0.68420	-1.36361
H	6.27121	-2.15083	-1.35514
C	7.88965	-2.14165	0.01009
O	7.55372	-2.80470	1.12964
H	6.61787	-2.67730	1.32781
O	9.02038	-2.17169	-0.38780
C	-2.64463	2.70657	-0.37365
H	-2.03392	2.15463	-1.10396
H	-3.32485	3.34322	-0.96788
C	-3.48960	1.71986	0.43531
H	-2.81995	1.06960	1.02334
H	-4.09028	2.27633	1.17559
C	-4.41232	0.86215	-0.42186
H	-3.81109	0.31049	-1.16576
H	-5.07961	1.51857	-1.00750
C	-5.25239	-0.12598	0.37832
H	-5.85292	0.42570	1.12267
H	-4.58410	-0.78176	0.96348
C	-6.17662	-0.98423	-0.47725
H	-6.84578	-0.32817	-1.06120
H	-5.57607	-1.53454	-1.22277
C	-7.01519	-1.97401	0.32227
H	-7.61566	-1.42405	1.06829
H	-6.34619	-2.63066	0.90610
C	-7.94047	-2.83243	-0.53279
H	-8.60763	-2.17454	-1.11553
H	-7.33896	-3.38015	-1.27828
C	-8.77426	-3.81848	0.27226
H	-9.41544	-3.29896	1.00088
H	-9.42876	-4.41965	-0.37532
H	-8.13567	-4.51532	0.83667

Table S11. Geometry of oleate in the orthogonal Cartesian coordinate format optimized in the gas phase at DFT level.

C	-1.62878	2.39118	-0.02305
H	-2.17516	3.34070	0.00065
C	-0.29130	2.42671	-0.03734
H	0.19984	3.40597	-0.02439
C	0.60542	1.22175	-0.07006
H	0.36274	0.60616	-0.95612
H	0.37634	0.56886	0.79255
C	2.09496	1.54811	-0.07474
H	2.32157	2.19226	-0.94215
H	2.33534	2.15428	0.81598
C	2.99423	0.31925	-0.10837
H	2.74672	-0.28630	-0.99875
H	2.76081	-0.32404	0.75886
C	4.48559	0.63430	-0.11331
H	4.73626	1.23492	0.77872
H	4.71788	1.28115	-0.97806
C	5.38566	-0.59617	-0.15174
H	5.13969	-1.19405	-1.04913
H	5.14336	-1.24671	0.70877
C	6.87617	-0.27689	-0.13450
H	7.14745	0.30083	0.76424
H	7.11494	0.38683	-0.98534
C	7.79980	-1.48185	-0.16216
H	7.76275	-2.01241	-1.12817
H	7.47034	-2.21479	0.59912
C	9.30093	-1.14207	0.16143
O	9.47823	-0.12484	0.86324
O	10.13198	-1.94670	-0.29513
C	-2.45331	1.13407	-0.03816
H	-2.16280	0.48948	0.81158
H	-2.20600	0.53915	-0.93668
C	-3.95692	1.37968	0.00574
H	-4.20189	1.96548	0.90887
H	-4.24496	2.01637	-0.84897
C	-4.78834	0.10372	-0.01100
H	-4.49558	-0.53243	0.84264
H	-4.53907	-0.48085	-0.91396
C	-6.29260	0.34346	0.03327
H	-6.58545	0.98004	-0.82016
H	-6.54219	0.92650	0.93720
C	-7.12145	-0.93533	0.01463
H	-6.87142	-1.51781	-0.88945
H	-6.82733	-1.57215	0.86731
C	-8.62613	-0.69900	0.05966
H	-8.92101	-0.06158	-0.79268
H	-8.87718	-0.11771	0.96456
C	-9.45388	-1.97897	0.03935
H	-9.20140	-2.55834	-0.86516
H	-9.15676	-2.61522	0.89046
C	-10.95557	-1.73714	0.08535
H	-11.29086	-1.13485	-0.77306
H	-11.51925	-2.68127	0.06747
H	-11.24633	-1.19502	0.99835

Table S12. Geometry of oleate in the orthogonal Cartesian coordinate format optimized in water at IEF-PCM DFT level.

C	-1.74573	3.58536	0.43566
H	-2.29709	4.20235	1.15602
C	-0.41109	3.70924	0.42248
H	0.03119	4.41867	1.13288
C	0.57780	2.98746	-0.44131
H	1.11477	3.72801	-1.06137
H	0.06729	2.31467	-1.14671
C	1.61070	2.19522	0.36371
H	2.11042	2.87102	1.07938
H	1.08888	1.44127	0.97750
C	2.66116	1.50979	-0.50148
H	3.17663	2.26886	-1.11586
H	2.15933	0.83677	-1.21873
C	3.69386	0.71891	0.29269
H	3.17806	-0.04035	0.90665
H	4.19416	1.39219	1.01099
C	4.74777	0.03478	-0.57030
H	5.26024	0.79488	-1.18683
H	4.24700	-0.63891	-1.28854
C	5.78290	-0.75248	0.22457
H	5.27749	-1.51394	0.84139
H	6.28193	-0.08460	0.94623
C	6.83816	-1.43163	-0.63079
H	7.36821	-0.68793	-1.25172
H	6.36395	-2.11526	-1.35724
C	7.91556	-2.24551	0.12935
O	7.83085	-2.29140	1.37682
O	8.78445	-2.78987	-0.58877
C	-2.60132	2.69255	-0.41049
H	-1.99019	2.13002	-1.13222
H	-3.28278	3.32037	-1.01266
C	-3.44665	1.71742	0.41227
H	-2.77725	1.07254	1.00676
H	-4.04634	2.28435	1.14542
C	-4.37089	0.85017	-0.43361
H	-3.77027	0.28846	-1.17033
H	-5.03797	1.50063	-1.02609
C	-5.21141	-0.12677	0.37980
H	-5.81078	0.43521	1.11746
H	-4.54311	-0.77664	0.97154
C	-6.13769	-0.99409	-0.46443
H	-6.80655	-0.34398	-1.05544
H	-5.53818	-1.55487	-1.20295
C	-6.97715	-1.97250	0.34816
H	-7.57650	-1.41206	1.08735
H	-6.30841	-2.62323	0.93893
C	-7.90449	-2.83998	-0.49551
H	-8.57125	-2.18809	-1.08551
H	-7.30412	-3.39819	-1.23413
C	-8.73924	-3.81458	0.32263
H	-9.37885	-3.28437	1.04508
H	-9.39526	-4.42281	-0.31700
H	-8.10070	-4.50509	0.89500

Table S13. Geometry of oleic acid in the orthogonal Cartesian coordinate format optimized in n-pentadecane at IEF-PCM DFT level.

C	-1.73970	3.53987	0.42540
H	-2.27934	4.17302	1.14046
C	-0.40395	3.64168	0.40660
H	0.05105	4.35305	1.10675
C	0.57115	2.89479	-0.45055
H	1.07122	3.61131	-1.12769
H	0.05108	2.17770	-1.10359
C	1.64686	2.16492	0.35707
H	2.17536	2.89200	0.99807
H	1.16107	1.45622	1.04911
C	2.65942	1.42246	-0.50642
H	3.11551	2.13028	-1.22130
H	2.13057	0.67657	-1.12599
C	3.76190	0.72920	0.28552
H	3.30798	0.03874	1.01802
H	4.30474	1.48040	0.88604
C	4.75845	-0.03880	-0.57577
H	5.18820	0.64657	-1.32906
H	4.21667	-0.80809	-1.15577
C	5.88609	-0.69724	0.21099
H	5.47447	-1.37744	0.97384
H	6.43181	0.06971	0.78625
C	6.87655	-1.47362	-0.63898
H	7.34845	-0.81831	-1.39154
H	6.35053	-2.24928	-1.22542
C	8.02061	-2.19359	0.14343
O	7.91957	-2.20983	1.38838
O	8.91203	-2.69254	-0.57271
C	-2.61016	2.65273	-0.41142
H	-2.00612	2.07142	-1.12410
H	-3.27668	3.28522	-1.02591
C	-3.47614	1.70141	0.41703
H	-2.82066	1.05226	1.02161
H	-4.06929	2.28554	1.14228
C	-4.41088	0.84244	-0.42574
H	-3.81582	0.26424	-1.15408
H	-5.06292	1.49840	-1.02925
C	-5.27356	-0.11368	0.38904
H	-5.86760	0.46323	1.11960
H	-4.62076	-0.77072	0.98969
C	-6.20901	-0.97011	-0.45618
H	-6.86211	-0.31243	-1.05659
H	-5.61417	-1.54506	-1.18738
C	-7.07116	-1.92962	0.35502
H	-7.66558	-1.35570	1.08790
H	-6.41827	-2.58874	0.95386
C	-8.00731	-2.78476	-0.49150
H	-8.65816	-2.12437	-1.08988
H	-7.41141	-3.35619	-1.22348
C	-8.86470	-3.74153	0.32408
H	-9.50041	-3.19813	1.04013

H	-9.52633	-4.34129	-0.31774
H	-8.24263	-4.44055	0.90398

Table S14. Geometry of the oleic acid·L-Arg adduct in the orthogonal Cartesian coordinate format optimized in the gas phase at DFT level.

C	-10.49735	1.39060	-0.24579
C	-11.91627	1.73539	0.19400
N	-12.90609	0.66889	0.09422
C	-9.81079	0.35978	0.63757
C	-8.41005	0.01561	0.15548
N	-7.78193	-0.94417	1.03146
C	-6.45569	-1.25868	1.00779
N	-5.63628	-0.65297	0.16690
N	-5.98659	-2.16935	1.84528
C	-12.43623	2.97425	-0.56425
O	-11.73167	3.89626	-0.86780
O	-13.73657	2.92117	-0.80414
H	-11.88771	2.05651	1.24980
H	-10.52756	1.03600	-1.29117
H	-9.92417	2.32873	-0.26401
H	-9.74802	0.73339	1.67300
H	-10.41681	-0.56321	0.67321
H	-8.45951	-0.35306	-0.88741
H	-7.80111	0.93384	0.14143
H	-8.38736	-1.60056	1.50426
H	-6.58628	-2.49557	2.58855
H	-4.91046	-2.43142	1.83926
H	-12.72876	0.06195	-0.70373
H	-12.95393	0.08718	0.92436
H	-14.00616	2.03050	-0.47600
H	-4.55805	-0.87476	0.19816
H	-6.01655	-0.07077	-0.56271
O	-3.12739	-1.19981	0.25535
O	-3.49395	-2.75350	1.81996
C	-2.75016	-2.10198	1.04384
C	-1.26601	-2.43737	1.08820
H	-0.90703	-2.13629	2.08751
H	-1.18172	-3.53576	1.07656
C	-0.42135	-1.80001	-0.00134
H	-0.57139	-0.70944	0.02392
H	-0.79984	-2.11943	-0.98618
C	1.06252	-2.12758	0.11047
H	1.20059	-3.22337	0.09585
H	1.43806	-1.79663	1.09517
C	1.91302	-1.50050	-0.98772
H	1.54147	-1.83709	-1.97148
H	1.77099	-0.40576	-0.97711
C	3.39965	-1.81656	-0.87623
H	3.54128	-2.91175	-0.87697
H	3.77324	-1.47059	0.10365
C	4.24504	-1.20016	-1.98399
H	3.87938	-1.55473	-2.96337
H	4.10514	-0.10607	-1.99324
C	5.73891	-1.51035	-1.85971
H	5.86646	-2.60731	-1.82774
H	6.11134	-1.13485	-0.89461
C	6.54911	-0.96527	-2.99554
H	6.29012	-1.36974	-3.98144
C	7.51939	-0.04323	-2.95258
C	8.06742	0.66740	-1.75310
H	7.92044	1.75435	-1.88714
H	7.50660	0.39702	-0.84572
C	9.55816	0.40475	-1.52768
H	10.11568	0.65728	-2.44641
H	9.71321	-0.67642	-1.37356
C	10.14271	1.17949	-0.35308
H	9.98362	2.26023	-0.51443
H	9.58043	0.93117	0.56416
C	11.62582	0.91816	-0.11973
H	11.78349	-0.16197	0.04581
H	12.18773	1.16214	-1.03837
C	12.21250	1.69792	1.05089
H	11.64852	1.45626	1.96891
H	12.05662	2.77816	0.88345
C	13.69463	1.43484	1.28773
H	13.85057	0.35496	1.45829
H	14.25896	1.67376	0.36900
C	14.28214	2.21792	2.45628
H	13.71454	1.98042	3.37222
H	14.12670	3.29650	2.28308
C	15.76135	1.94797	2.68977
H	16.15094	2.52920	3.53799
H	15.94516	0.88411	2.90490
H	16.36103	2.21081	1.80478
H	7.99221	0.22798	-3.90423

Table S15. Geometry of the oleic acid·L-Arg adduct in the orthogonal Cartesian coordinate format optimized in water at IEF-PCM DFT level.

C	-10.82760	0.76648	0.59086
C	-12.21309	0.57200	1.19886
N	-13.12173	-0.30336	0.46881
C	-9.98840	-0.50261	0.58233
C	-8.62377	-0.28690	-0.05180
N	-7.84808	-1.50929	-0.02668
C	-6.52332	-1.58572	-0.24643
N	-5.81704	-0.49550	-0.51894
N	-5.90662	-2.76058	-0.17914
C	-12.92238	1.91934	1.41430
O	-12.37264	2.91356	1.82267
O	-14.20994	1.86009	1.13616
H	-12.09367	0.16112	2.21566
H	-10.93983	1.15406	-0.43620
H	-10.31937	1.55476	1.16469
H	-9.85284	-0.86764	1.61317
H	-10.51923	-1.29744	0.03266
H	-8.74800	0.08073	-1.08601
H	-8.08290	0.49260	0.50814
H	-8.35442	-2.38278	0.03852
H	-6.42002	-3.57251	0.13203
H	-4.86666	-2.83210	-0.37184
H	-12.91098	-0.32819	-0.52721
H	-13.11444	-1.25915	0.81138
H	-14.31173	0.92178	0.79566
H	-4.76883	-0.56298	-0.65558
H	-6.28920	0.37903	-0.69221
O	-3.16764	-0.69349	-0.86994
O	-3.28001	-2.91558	-0.66441
C	-2.65781	-1.84106	-0.85836
C	-1.15908	-1.96487	-1.12959
H	-0.76642	-2.75274	-0.46756
H	-1.05895	-2.37189	-2.15154
C	-0.35307	-0.68356	-0.99714
H	-0.45503	-0.29299	0.02953
H	-0.79560	0.08538	-1.64970
C	1.12386	-0.85796	-1.32956
H	1.22265	-1.23986	-2.36116
H	1.55634	-1.64027	-0.68105
C	1.94206	0.41991	-1.18671
H	1.50642	1.20458	-1.82990
H	1.85028	0.79678	-0.15292
C	3.41674	0.24810	-1.53048
H	3.50910	-0.12058	-2.56716
H	3.84943	-0.54289	-0.89311
C	4.23843	1.52204	-1.37653
H	3.80823	2.31494	-2.01271
H	4.15626	1.89115	-0.34001
C	5.71705	1.33956	-1.72767
H	5.78339	0.96112	-2.76362
H	6.15134	0.55534	-1.08949
C	6.50693	2.60898	-1.62894
H	6.16029	3.41881	-2.28278
C	7.55774	2.87009	-0.83870
C	8.23229	1.95303	0.13569
H	8.20195	2.41662	1.13799
H	7.68640	1.00162	0.22358
C	9.69413	1.67009	-0.21998
H	10.23558	2.62642	-0.32353
H	9.73709	1.19183	-1.21334
C	10.40920	0.79207	0.79966
H	10.36463	1.27621	1.79098
H	9.86138	-0.16043	0.90781
C	11.86244	0.49898	0.44643
H	11.90536	0.01534	-0.54523
H	12.41004	1.45159	0.33757
C	12.57911	-0.37931	1.46522
H	12.02993	-1.33092	1.57538
H	12.53751	0.10522	2.45658
C	14.03144	-0.67565	1.11129
H	14.07331	-1.16071	0.12000
H	14.58118	0.27578	1.00062
C	14.74886	-1.55398	2.13013
H	14.19736	-2.50335	2.24045
H	14.70572	-1.06803	3.11988
C	16.19817	-1.84650	1.76962
H	16.68373	-2.48128	2.52508
H	16.27220	-2.36646	0.80198
H	16.78513	-0.91853	1.68892
H	8.00246	3.87012	-0.91353

Table S16. Geometry of the oleic acid·L-Arg adduct in the orthogonal Cartesian coordinate format optimized in n-pentadecane at IEF-PCMPDFT level.

C	-10.59747	-1.32603	0.00378
C	-12.02896	-1.54211	-0.47602
N	-12.97204	-0.46146	-0.21458
C	-9.87241	-0.20124	-0.72013
C	-8.45446	0.00035	-0.20984
N	-7.79952	1.07420	-0.92180
C	-6.47828	1.36378	-0.83114
N	-5.67506	0.61743	-0.09053
N	-5.98361	2.39711	-1.49701
C	-12.60878	-2.85056	0.09485
O	-11.96234	-3.85467	0.23356
O	-13.89640	-2.75689	0.37296
H	-12.01102	-1.70791	-1.56702
H	-10.61275	-1.13062	1.09024
H	-10.05992	-2.27618	-0.12615
H	-9.83630	-0.41389	-1.80125
H	-10.43385	0.74210	-0.60309
H	-8.47696	0.19675	0.87838
H	-7.88407	-0.93109	-0.35759
H	-8.38973	1.77264	-1.35219
H	-6.57606	2.89000	-2.14838
H	-4.92624	2.63407	-1.43541
H	-12.74806	0.04059	0.64245
H	-13.02438	0.21594	-0.96856
H	-14.10582	-1.80524	0.19217
H	-4.61434	0.82760	-0.06508
H	-6.06240	-0.10639	0.49447
O	-3.12076	1.15193	-0.04463
O	-3.44509	2.94500	-1.33908
C	-2.72155	2.16544	-0.66930
C	-1.23104	2.48610	-0.63665
H	-0.86286	2.37515	-1.67120
H	-1.13471	3.56156	-0.41617
C	-0.40114	1.64813	0.32041
H	-0.55396	0.58286	0.08616
H	-0.78951	1.77646	1.34418
C	1.08540	1.98121	0.28835
H	1.22818	3.05139	0.52148
H	1.46808	1.84973	-0.73945
C	1.92324	1.14297	1.24612
H	1.54359	1.27632	2.27414
H	1.77977	0.07333	1.01367
C	3.41191	1.46953	1.21330
H	3.55638	2.53927	1.44617
H	3.79149	1.33608	0.18497
C	4.24851	0.62977	2.17076
H	3.87382	0.76583	3.20016
H	4.11011	-0.44054	1.94236
C	5.74253	0.96011	2.12923
H	5.86794	2.03627	2.34604
H	6.12226	0.81126	1.10715
C	6.54660	0.17364	3.11871
H	6.26454	0.32793	4.16735
C	7.53819	-0.69393	2.87644
C	8.12011	-1.08233	1.55313
H	7.99768	-2.17759	1.42183
H	7.56628	-0.61905	0.72555
C	9.60749	-0.74935	1.42652
H	10.15807	-1.20801	2.26622
H	9.74027	0.33962	1.54235
C	10.22518	-1.20289	0.10949
H	10.08686	-2.29277	-0.00132
H	9.67088	-0.74656	-0.72932
C	11.70561	-0.86674	-0.02405
H	11.84293	0.22308	0.08742
H	12.25993	-1.32309	0.81477
C	12.32425	-1.31965	-1.34122
H	11.76953	-0.86331	-2.17983
H	12.18642	-2.40955	-1.45253
C	13.80456	-0.98395	-1.47593
H	13.94287	0.10606	-1.36494
H	14.35983	-1.44011	-0.63735
C	14.42369	-1.43699	-2.79332
H	13.86708	-0.98103	-3.62986
H	14.28387	-2.52604	-2.90271
C	15.90178	-1.09836	-2.92093
H	16.31453	-1.43903	-3.88156
H	16.07082	-0.01260	-2.85385
H	16.49128	-1.57133	-2.12045
H	8.00320	-1.17542	3.74532
