# Supplementary material for: Effect of chemical interaction between oleic acid and L-Arginine on oral perception, as a function of polymorphisms of CD36 and OBPIIa and genetic ability to taste 6-n-propylthiouracil
Source: PLoS One. 2018 Mar 22;13(3):e0194953. doi: 10.1371/journal.pone.0194953 (PMC5864069; doi:10.1371/journal.pone.0194953)
Supplement: S2 File — (PDF) [file pone.0194953.s003.pdf]

S2 File. Molecular analysis at polymorphisms *rs713598* of *TAS2R38* locus and *rs1761667* of *CD36* gene.

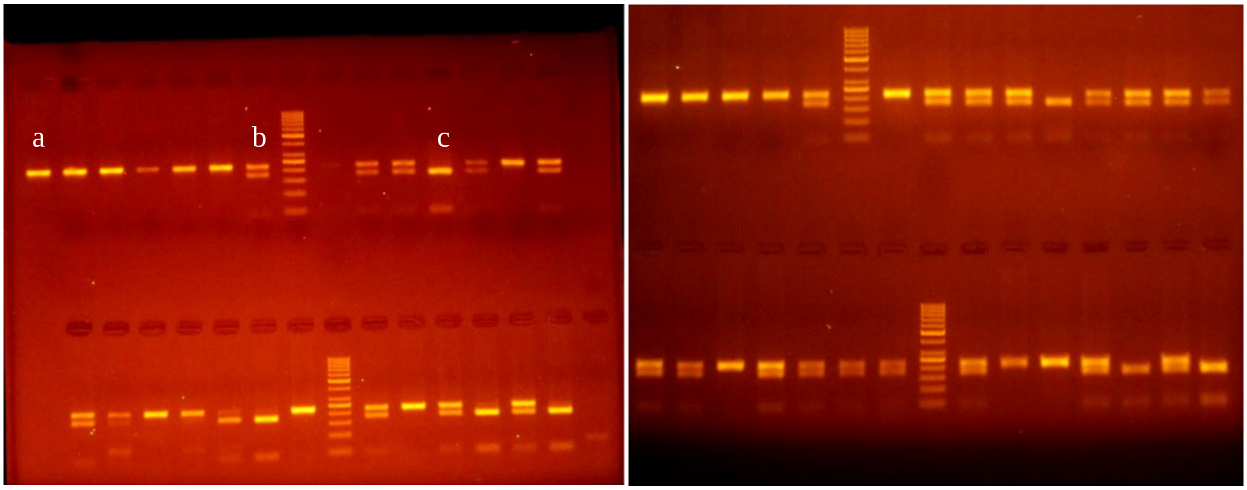

Photographs of DNA bands separated by electrophoresis in agarose gel following the digestion with restriction enzyme (*HaeIII*) of the fragments including *rs713598* of *TAS2R38* locus. Allele sizes: 241 bp = A/A homozygous (a); 241, 195, 46 bp = heterozygous (b); 195, 46 bp = P/P homozygous (c). P/P homozygous (n=8), heterozygous (n=22) and A/A homozygous (n=16). 4 controls heterozygous and 4 A homozygous are included.

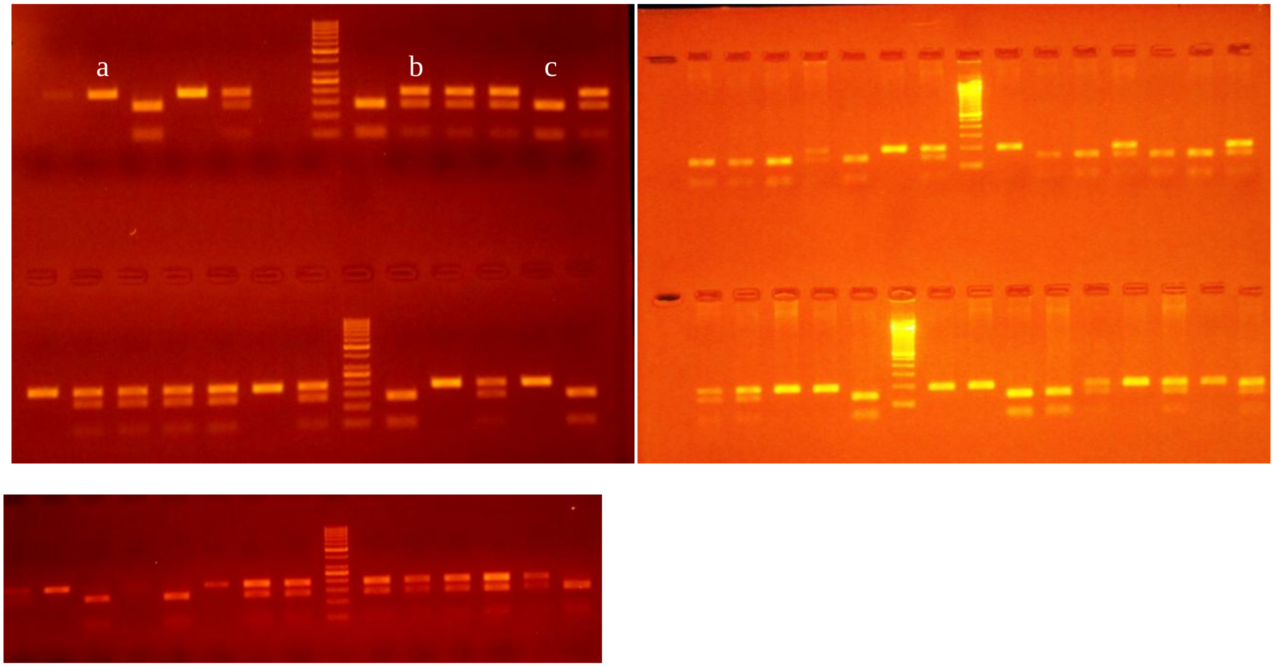

Photographs of DNA bands separated by electrophoresis in agarose gel following the digestion with restriction enzyme (*Hha I*) of the fragments including *rs1761667* of *CD36* locus. Allele sizes: 190 bp = AA homozygous (a); 190, 138, 52 bp = heterozygous (b); 138, 52 bp = GG homozygous (c). GG homozygous (n=15), heterozygous (n=23) and A homozygous (n=8). 8 A homozygous, 2 controls heterozygous and 4 G homozygous are included.
